# Exploring Biomarkers for Excess Extracellular Fluid in the Context of Physical Function in Chronic Kidney Disease Patients

**DOI:** 10.3390/jpm14121124

**Published:** 2024-11-27

**Authors:** Hyae Min Lee, Jihyun Baek, So-Young Lee, Yu Ho Lee, Sang Hyun Jung, Hye Yun Jeong

**Affiliations:** Division of Nephrology, Department of Internal Medicine, CHA Bundang Medical Center, CHA University School of Medicine, Seongnam 13496, Republic of Korea; ihyaemin@gmail.com (H.M.L.); spreesh7@chamc.co.kr (J.B.); ysy0119@cha.ac.kr (S.-Y.L.); borywork@chamc.co.kr (Y.H.L.); sd2527@chamc.co.kr (S.H.J.)

**Keywords:** BDNF, biomarker, chronic kidney disease, fluid overload, physical function

## Abstract

**Background/Objectives**: Fluid overload is an important risk factor for protein-energy wasting, which could lead to poor outcomes, such as higher morbidity and mortality, in patients with chronic kidney disease (CKD). This study aimed to validate the possible myokine as a biomarker of volume status in patients with non-dialysis CKD. **Methods**: In total, 151 patients with CKD were enrolled from a single medical center. Demographic data were collected via medical record review. Bioimpedance analysis was performed to measure body composition, and physical performance was assessed by measuring hand grip strength. **Results**: The physical performance of hand grip strength (21.9 ± 8.7 vs. 19.0 ± 10.1 kg, *p* = 0.233) and walking speed (1.05 ± 0.29 vs. 0.86 ± 0.52 m/s, *p* = 0.192) was higher in the low extracellular water/total body water (ECW/TBW) ratio group. Although higher, the median value of the brain-derived neurotrophic factor (BDNF) was not significant in the low ECW/TBW ratio group. Despite that, it had significant negative correlations with the ECW/TBW ratio in Pearson’s correlation analyses (r = −0.329, *p* < 0.001; r = −0.287, *p* < 0.001; and r = −0.238, *p* = 0.003). Linear regression analysis showed that the BDNF level had a significant negative relationship with the ECW/TBW ratio and significant associations even after multivariate analysis. **Conclusions**: Among myokines, BDNF had a significant negative relationship with the ECW/TBW ratio, suggesting that BDNF could be a possible biomarker for volume status in patients with non-dialysis CKD.

## 1. Introduction

Fluid overload, an abnormal hydration status, is a common phenomenon in patients with chronic kidney disease (CKD), which reportedly causes adverse outcomes, particularly in the late stages of the disease [1]. Given that fluid overload is related to hypertension, congestive heart failure, left ventricular hypertrophy, and pulmonary edema, it is also closely related to cardiovascular morbidity and survival outcomes in these patients [2,3]. In these respects, exploring volume status in patients with CKD is crucial, playing a key role in managing their overall health and preventing complications.

However, assessing the volume status in patients with CKD is complex and challenging, for several reasons, mainly due to the complex interactions between kidney function, fluid balance, and the body’s compensatory mechanisms. Bioimpedance spectroscopy (BIS) is an increasingly used tool to detect volume overload, and bioimpedance-defined extracellular water/total body water (ECW/TBW) ratio is a well-validated predictor of survival in patients on hemodialysis (HD) [4,5]. However, BIS analysis differs in accuracy and validity between machines and demonstrates a higher bias in fluid overload status [5,6]. In addition, changes were observed in the extreme body mass index (BMI) range, altered fat/lean mass ratios, inaccurately self-reported height and weight, and patients with acute illness necessitate BIS validation [7]. Therefore, the difficulty with existing volume measurement tools highlights the need for more accurate, reliable, and accessible biomarkers to predict volume status. Using readily available laboratory biomarkers and combining these tools might enhance their accuracy in assessing volume status and improving outcomes in patients with CKD.

Among the variable outcomes related to the volume status of patients with CKD, fluid overload is a crucial risk factor for protein-energy wasting and malnutrition, eventually leading to poor outcomes [8]. Decreased body protein storage in protein-energy wasting is associated with decreased functional capacity and physical function, which increases mortality in CKD [9,10]. Although studies on the direct relationship between volume status and physical function in patients with CKD are rare, Hsiao et al. previously reported a significant association between high fluid status and impaired extremity muscle endurance [11].

We took an approach and investigated the relationship between volume status, estimated as ECW/TBW, and known myokines that could reflect muscular function to explore the possible biomarkers related to volume status in the context of physical function. Among various myokines, the brain-derived neurotrophic factor (BDNF) has been suggested as a myokine that reprograms skeletal muscle [12] and is a possible biomarker of functional capacity in patients with chronic obstructive pulmonary disease (COPD) [13] and in hemodialysis patients [14]. In addition, we also used known myokines including myostatin, osteocalcin, and Matrix Metalloproteinase-9 (MMP-9).

This study aimed to evaluate the possible myokine as a biomarker to reflect the volume status in patients with non-dialysis CKD.

## 2. Materials and Methods

### 2.1. Patients 

One hundred and fifty-one patients with CKD were recruited from the CHA Bundang Medical Center over six months starting in June 2016. The inclusion criteria were adult patients aged >20 years with a confirmed diagnosis of CKD. CKD was defined as having two times the previously estimated glomerular filtration rate (eGFR) values <60 mL/min/1.73 m^2^ calculated by the equation of the Modification of Diet in Renal Disease Study Group; eGFR was obtained at 3–6-month intervals [15]. Patients with a history of active infection, coagulation disorders, cancer, or kidney transplantation, and those unable to ambulate were excluded. This study was approved by the Institutional Review Board of CHA Bundang Medical Center and was it was performed in accordance with the Declaration of Helsinki and the principles of Good Clinical Practice (approval code: CHAMC 2016-05-064-024, approval date: 16 June 2015).

### 2.2. Clinical Variables

The demographic and clinical data of the patients, including age, sex, etiology of CKD, blood pressure, and comorbidities, were obtained through a medical record review. Cardiovascular disease was defined by a medical history of angina pectoris, myocardial infarction, percutaneous transluminal coronary angioplasty, congestive heart failure, or coronary artery bypass. Laboratory findings, including creatinine, albumin, intact parathyroid hormone (iPTH), serum calcium, phosphorus, albumin, low-density lipid (LDL) cholesterol, and high-sensitivity C-reactive protein (hs-CRP), were collected at the time of patient enrollment. Estimated GFR (eGFR) was calculated based on Modification of Diet in Renal Disease (MDRD) equation.

### 2.3. Body Composition Measurement

Bioimpedance analysis (Inbody 620, In-body, Seoul, Republic of Korea) was performed at 5, 50, and 500 kHz to assess body composition. The results of body composition analysis included appendicular lean mass (ALM), body mass index (BMI), fat free mass (FFM), extracellular water (ECW), intracellular water (ICW), and total body water (TBW). ALM was defined as the total mass of both arms and legs excluding fat and bone, as measured by DXA. Weight-adjusted ALM (ALM/weight) and BMI-adjusted ALM (ALM/BMI) were evaluated in all subjects. We defined an ECW/TBW ≥ 0.400 as the high ECW/TBW group [16,17].

### 2.4. Grip Strength and Physical Performance

A Jamar hand dynamometer (Sammons Preston Inc., Bolingbrook, IL, USA) was used to measure the maximum hand grip strength. The arm holding the dynamometer was positioned with the elbow resting against the body and bent at a 90° angle, while the forearm was supported on an armrest. Participants were asked to grip the dynamometer handle with maximum force for 3 s. The average value of the three tests performed was used for analysis. Walking speed was calculated based on the time to walk 4 m as suggested by the Asian Working Group for Sarcopenia (AWGS) [18]. A 4-m straight path was designated for the participant to walk. The participant was instructed to walk at their normal, comfortable pace from the beginning to the end of the 4 m track. The walking speed was measured by recording the time it took to traverse the 4 m distance.

### 2.5. Laboratory Measurements

Plasma samples for BDNF, myostatin, osteocalcin, and Matrix Metalloproteinase-9 (MMP-9) were collected in ethylenediaminetetraacetic acid-treated tubes during patient enrollment. The samples were stored at −80 °C after centrifugation for 15 min at 1000× *g* at room temperature. Magnetic Luminex^®^ Screening Assay multiplex kits (R&D Systems, Inc., Minneapolis, MN, USA) were used to perform enzyme-linked immunosorbent assay.

### 2.6. Statistical Analysis

Categorical variables were presented as numbers and percentages. Continuous variables were recorded as the mean ± standard variation or median (IQR). Student’s *t*-test and Mann–Whitney *U* test were used to compare continuous variables. Categorical variables were analyzed using χ2 tests or Fisher’s exact tests. Pearson’s correlation coefficients were used to analyze the cross-sectional relationships among the variables. Univariate and multivariate linear regression analyses were used to investigate the associations between BDNF levels and possible associated factors. Statistical significance was set at *p* < 0.05. Statistical analyses were performed using IBM SPSS Statistics for Windows version 21 (IBM Corp., Armonk, NY, USA).

## 3. Results

### Clinical and Laboratory Characteristics of Patients

**Figure 1:**
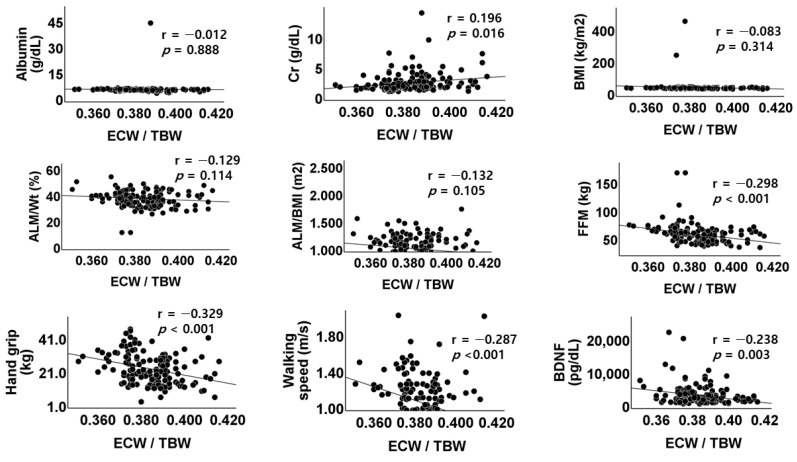
Scatterplots and correlations of ECW/TBW versus albumin, creatinine, BMI, weight-adjusted appendicular lean mass (ALM), body mass index-adjusted ALM, fat-free mass, handgrip strength, walking speed, or BDNF level. ALM, appendicular lean mass; BMI, body mass index; ALM/wt, weight-adjusted ALM; ALM/BMI, body mass index-adjusted ALM; FFM, fat-free mass; BDNF, brain-derived neurotrophic factor; r, Pearson’s correlation coefficient; *p*, *p*-value.

**Figure 2:**
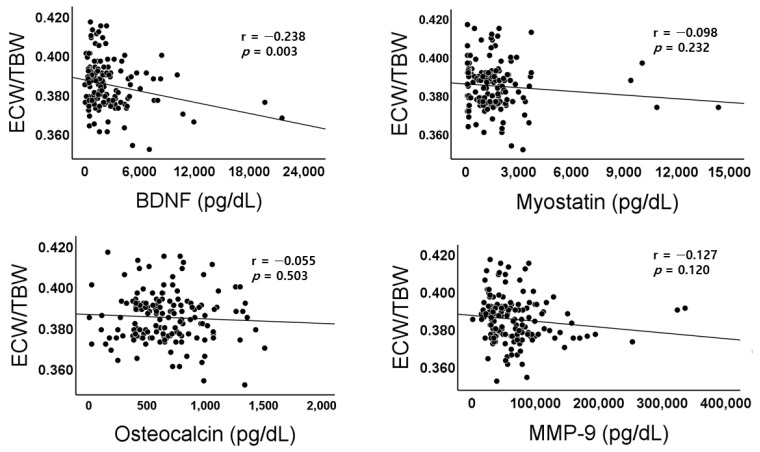
Scatterplots and correlations of ECW/TBW versus BDNF, Myostatin, Osteocalcin, or MMP-9. BDNF, brain-derived neurotrophic factor; MMP-9, Matrix Metalloproteinase-9; r, Pearson’s correlation coefficient; *p*, *p*-value.

The baseline clinical and biochemical findings are shown in Table 1. The average age of total subjects was 64.8 ± 13.2 year. Among patients recruited during the study periods, there were 136 subjects (90.0%) whose ECW/TBW ratio was lower than 0.400. The serum creatinine (2.09 ± 1.63 vs. 2.78 ± 1.68, *p* = 0.479), calcium (9.03 ± 0.60 vs. 8.88 ± 0.44 mg/dL, *p* = 0.353), phosphorus (3.56 ± 0.63 vs. 3.36 ± 0.53 mg/dL, *p* = 0.253), and albumin level were not different between low and high ECW/TBW ratio group. The physical performance of hand grip strength (21.9 ± 8.7 vs. 19.0 ± 10.1 kg, *p* = 0.233) and walking speed (1.05 ± 0.29 vs. 0.86 ± 0.52 m/s, *p* = 0.192) were not different between the two groups; however, they had significant negative correlations with ECW/TBW ratio in Pearsons’s correlation analyses (r = −0.329, *p* < 0.001; r = −0.287, *p* < 0.001; r = −0.238, *p* = 0.003) (Figure 1). In the measurement of the myokine levels, which are known to be involved in exercise-associated metabolic changes, the differences were not significant between the two groups in all four myokines. Among them, the median value of BDNF was not significantly different between the two groups, but a higher value was measured in the low ECW/TBW ratio group (1670.70 (820.29–3328.72) vs. 1148.99 (610.36–1740.20), *p* = 0.052). In the Pearson’s correlation analysis, only BDNF had a significant correlation with ECW/TBW ratio (r = −0.238, *p* = 0.003), among the four myokines (Figure 2).

We also compared the biochemical findings, body composition, and physical performance between the low and high BDNF levels based on median values (Table 2). The age (67.70 ± 11.56 vs. 61.76 ± 14.07, *p* = 0.005), BMI (24.25 (22.00–28.10) vs. 24.60 (21.90–26.60) kg/m^2^, *p* = 0.558) and BMI-adjusted ALM (0.97 ± 0.24 vs. 0.98 ± 0.26, *p* = 0.692) were not different between the two groups. Although not significant, the physical performance for hand grip strength (20.7 ± 9.4 vs. 22.6 ± 8.3 kg, *p* = 0.182) and walking speed (0.99 ± 0.36 vs. 1.07 ± 0.27 m/s, *p* = 0.147) was higher in the high BDNF group. The ECW/TBW ratio was significantly higher in the low BDNF group (0.386 ± 0.012 vs. 0.382 ± 0.012 pg/mL, *p* = 0.048).

We used a linear regression analysis to determine possible factors associated with BDNF.

In the linear regression analysis, serum albumin, creatinine, and BMI levels were negatively correlated with the ECW/TBW ratio; however, these were not statistically significant. The BDNF level also had negative relation with ECW/TBW ratio (β = −0.238, 95% CI: −9.93 × 10^4^, −2.04 × 10^4^, *p* = 0.003), and it was statistically associated with ECW/TBW ratio even after multivariable analysis (β = −0.222, 95% CI: −1.07 × 10^5^, −8.61 × 10^3^, *p* = 0.022) (Table 3). These results show that BDNF level might be a possible biomarker reflecting the ECW/TBW ratio for volume status in patients with CKD.

## 4. Discussion

This study aimed to assess whether BDNF, a myokine, has any significant association with the ECW/TBW ratio, a well-known indicator of volume overload, and determine its use as a biomarker reflecting volume status in patients with non-dialysis CKD. Although the BDNF levels were low in the high ECW/TBW ratio group, Pearson’s correlation analysis showed a significant negative correlation. We additionally compared the variables between the two groups with high or low BDNF levels and found that the ECW/TBW ratio was significantly lower in the high BDNF group than in the low BDNF group. In the multivariate linear regression analysis, BDNF levels had a significant negative relationship with the ECW/TBW ratio, which remained significant even after adjusting for possible confounding factors.

This present study showed that physical performance was low in the patient group with high ECW/TBW ratios; however, the difference was not statistically significant. This relationship is similar to that reported in previous studies that investigated the association between the two parameters in the general population or patients with CKD. Water represents more than 70% of the main body component. Gradual intracellular dehydration occurs with age, eventually leading to adverse cellular results, particularly affecting muscle cells, such as muscle wasting, catabolism, impaired muscle contractility, and impaired cellular protein structure [19]. Low intracellular water (ICW) is significantly related to functional capacity and muscle strength [20,21,22], and increased ECW could predict gait speed and knee extension force [23]. In this regard, an imbalance in the ICW to ECW ratio may be reflected in the ECW/TBW ratio, which is reportedly a significant factor in nutritional status and an indicator of edema [24,25]. Additionally, this ratio is negatively correlated with serum albumin, and Ge et al. showed that the ECW/TBW ratio is a better indicator of survival in patients with sarcopenia than other indices [26].

Loss of hand grip strength in patients with heart failure is associated with muscle quality, as indicated by increased upper limb ECW/ICW [27]. In the general population, segmental BIS analysis for thigh and lower leg ECW/ICW reported the negative association between this ratio and gait speed and knee extension strength in older participants [23,28], and a cross-sectional study of community-dwelling women aged >65 years showed that the total body ECW/ICW ratio was negatively associated with gait speed and handgrip strength.

A recent study also showed a significant association between high fluid status and reduced upper extremity muscle endurance in patients with CKD. Fluid overload is suggested to be related to the malnutrition-inflammation status in patients with CKD, as inflammation inhibits water channels and interrupts transcellular water permeability. However, they showed that abnormal volume status was a potential indicator of upper extremity muscle strength in patients with CKD, independent of malnutrition-inflammation status [11,29].

Similarly, Rakase et al. suggested that an increase in the ECW/TBW ratio reflects knee extension strength in patients with CKD [30], and Seto et al. demonstrated that a significant decrease in muscle mass was related to an ECW/TBW increase in hemodialysis patents [31].

Sarcopenia, characterized by loss of muscle mass and/or muscle strength, is an essential disease seen in advanced age and patients with CKD and is strongly associated with mortality of these populations; various study groups have suggested the importance of assessing muscle mass and quality reflected by physical performance [32]. Following the recent revision of the 2019 consensus update from the AWGS, Park et al. investigated the relationship between sarcopenia and the ECW/TBW ratio, which is an easily measured value using the BIS. They demonstrated that sarcopenia and severe sarcopenia were significantly associated with a high ECW/TBW ratio and a much higher ECW/TBW ratio in the low-short physical performance battery group than in the control group. They conclusively suggested that ECW/TBW may be a valid marker for assessing physical function and sarcopenia [33].

Although BIS is a relatively convenient tool for assessing body composition, including volume status reflected by ICW, ECW, or TBW, it is time-consuming, and skin temperature, sweating, changes in electrolyte content, extremely high BMI, or positional changes could interfere with BIS [34]. As a blood biomarker could be a widely accessible and simple triage tool for a large population, developing plasma biomarkers for volume status and physical function would be a potential tool for screening and establishing therapeutic targets for candidate populations. The research focused on how discovering biomarkers that can accurately predict fluid status in CKD patients and integrating them with existing diagnostic methods could improve personalized treatment and outcomes for these patients.

BDNF is a neurotrophin family of growth factors that induces growth factor production related to differentiation and neural growth [35], which contributes to neuronal cell viability preservation and neuronal degradation prevention [36]. It also plays a pivotal role in non-neuronal cells, such as endothelial and smooth muscle cells [37,38]. BDNF expressed in the skeletal muscle regulates the survival of motor neurons and is involved in myoblast development and differentiation [35]. Recently, BDNF has been myokine that reprograms skeletal muscle [12] and a possible biomarker of functional capacity in patients with COPD [13]. Furthermore, a cross-sectional study of 20 Japanese hemodialysis patients showed that low BDNF levels were associated with decreased physical performance [14]. These data also suggest that BDNF levels could be a good marker reflecting volume status in parallel with the prediction of physical performance.

In addition, clinical trials based on a small number of patients with heart failure demonstrated low BDNF levels, and a study based on a larger population showed that low BDNF levels were associated with high levels of N-terminal pro-B-type natriuretic peptide (NT-proBNP), a well-known marker of fluid volume overload status and heart failure [39]. Despite the directional and causal relationship of BDNF with physical function or volume status, this study provides suggestive evidence of BDNF as a biomarker for volume status, which is significantly correlated with the ECW/TBW ratio.

The majority of circulating BDNF comes from megakaryocytes, and physical exercise activates platelets to release BDNF into the blood [40]. This means that alterations of BDNF levels induced by physical exercise might reflect changes in megakaryocytes and platelets, accompanied by production of BDNF in skeletal muscle. Miyazaki et al. suggests that proinflammatory status of chronic kidney disease patients would negatively affect the BDNF production in skeletal muscles, which indirectly results in impaired physical performances [14]. Looking at the content related to volume status, the low levels of BDNF could modulate left atrial remodeling that leads to atrial fibrillation [39], the strong risk factor of heart failure. In a large population-based cohort study, low BDNF had association with high incidences of cardiovascular events including heart failure [41]. Further research exploring corrected mechanism explaining BDNF’s role in muscle function and fluid regulation would be necessary.

There are some limitations in this study. First, the median values of BDNF were not significantly different between the high and low ECW/TBW ratio groups. However, they had significant correlations in the Pearson’s correlation analyses, although the correlations had relatively weak negative correlation. An analysis including a larger study population would be needed. Second, as this study was an observational cohort study showing the evidence of BDNF as a biomarker for volume status, it would be more convincing if we could provide further data on whether this translates into meaningful improvements in patient outcomes. Furthermore, providing clinical outcomes like heart function or nutritional status assessment would provide a more comprehensive view of the impact of fluid status on CKD patients. Third, we could not include a healthy control group. Further study using a control group could allow for statistical benchmarking of the laboratory outcomes, providing clearer insights into how the observed parameters differ from normal levels. Finally, it was difficult to explain the exact mechanism for how BDNF could be a biomarker for volume status.

## 5. Conclusions

BDNF has a significant negative relationship with the ECW/TBW ratio, and we might be able to suggest that it is a possible biomarker of volume status in patients with non-dialysis CKD. More studies may contribute to improving our understanding of the relationship between BDNF and volume overload, and it would be of interest to study whether this relationship is also applicable to other patients, such as those with dialysis-dependent CKD.

## Figures and Tables

**Table 1:** Baseline demographic and laboratory data of enrolled population according to the ECW/TBW ratio.

	Low ECW/TBW (*n* = 136)	High ECW/TBW (*n* = 15)	*p* Value
Age (year)	63.9 ± 13.3	71.0 ± 9.4	0.027
Male (*n*,%)	91 (66.9)	11 (73.3)	0.614
Hypertension (*n*,%)	105 (77.2)	12 (80.0)	0.551
Diabetes mellitus (*n*,%)	58 (42.6)	8 (53.3)	0.428
Previous history of CVD (*n*,%)	20 (14.7)	3 (20.0)	0.588
SBP (mmHg)	132.1 ± 14.6	134.3 ± 17.0	0.594
Hemoblobin (g/dL)	12.69 ± 3.58	11.76 ± 1.31	0.321
Creatinine (mg/dL)	2.10 ± 1.63	2.78 ± 1.68	0.127
eGFR (mL/min/1.73 m^2^)	43.96 ± 22.30	37.25 ± 23.30	0.560
iPTH (pg/dL)	50.0 (33.13–88.03)	69.35 (34.93–133.05)	0.377
Calcium (mg/dL)	9.03 ± 0.60	8.88 ± 0.44	0.353
Phosphorus (mg/dL)	3.56 ± 0.63	3.36 ± 0.53	0.253
Albumin (mg/dL)	4.52 ± 3.46	4.24 ± 0.40	0.758
LDL-cholesterol (mg/dL)	86.0 ± 36.9	84.8 ± 46.6	0.909
hs-CRP (mg/dL)	0.07 (0.03–0.15)	0.05 (0.03–0.12)	0.508
BMI (kg/m^2^)	24.30 (21.85–27.60)	25.65 (23.08–26.15)	0.457
ALM	23.93 ± 6.06	23.80 ± 7.47	0.939
ALM/Wt (%)	36.13 ± 6.09	35.89 ± 6.01	0.884
ALM/BMI (m^2^)	0.97 ± 0.24	1.01 ± 0.30	0.529
Hand grip (kg)	21.9 ± 8.7	19.0 ± 10.1	0.233
Walking speed (m/s)	1.05 ± 0.29	0.86 ± 0.52	0.192
BDNF (pg/mL)	1670.70 (820.29–3328.72)	1148.99 (610.36–1740.20)	0.052
Myostatin (pg/mL)	1449.96 (484.89–2207.14)	1450.89 (200.29–1784.63)	0.313
Osteocalcin (pg/mL)	669.93 ± 307.70	602.38 ± 291.20	0.419
MMP-9 (pg/mL)	68,218.61 ± 51,458.50	45,981.41 ± 22,705.84	0.101

**Abbreviation**: ALM, appendicular lean mass; ALM/BMI, BMI-adjusted ALM; ALM/Wt, weight-adjusted ALM; BDNF, brain-derived neurotrophic factor; BMI, body mass index; CVD, cardiovascular disease; ECW, extracellular water; eGFR, estimated glomerular filtration rate; hs-CRP, high-sensitivity C-reactive protein; iPTH, intact parathyroid hormone; LDL, low-density lipoprotein; MMP-9, Matrix Metalloproteinase-9; SBP, systolic blood pressure; TBW, total body water.

**Table 2:** Baseline demographic and laboratory data of study population according to the BDNF level.

	Low BDNF (*n* = 76)	High BDNF (*n* = 75)	*p* Value
Age (year)	67.70 ± 11.56	61.76 ± 14.07	0.005
Male (*n*,%)	11 (15.1)	12 (16.0)	0.794
Hemoblobin (g/dL)	12.81 ± 4.39	12.38 ± 2.04	0.446
Creatinine (mg/dL)	2.30 ± 1.94	2.03 ± 1.26	0.315
eGFR (mL/min/1.73 m^2^)	38.73 ± 20.46	48.26 ± 23.10	0.058
iPTH (pg/dL)	55.12 (25.83–88.59)	47.10 (31.00–112.2)	0.272
Calcium (mg/dL)	8.99 ± 0.53	9.05 ± 0.64	0.506
Phosphorus (mg/dL)	3.54 ± 0.69	3.54 ± 0.57	0.929
Albumin (mg/dL)	4.77 ± 4.65	4.23 ± 0.51	0.327
hs-CRP (mg/dL)	0.05 (0.03–0.17)	0.08 (0.03–0.12)	0.909
BMI (kg/m^2^)	24.25 (22.00–28.10)	24.60 (21.90–26.60)	0.558
ALM/Wt (%)	36.28 ± 5.97	35.93 ± 6.19	0.731
ALM/BMI (m^2^)	0.97 ± 0.24	0.98 ± 0.26	0.692
FFM (kg)	55.00 (49.28–64.35)	55.30 (45.30–62.60)	0.844
ICW	24.80 (22.30–29.23)	24.10 (20.10–27.90)	24.10 (20.10–27.90)
ECW	15.95 (13.95–18.13)	15.30 (12.80–17.70)	0.924
ECW/TBW	0.386 ± 0.012	0.382 ± 0.012	0.048
Hand grip (kg)	20.7 ± 9.4	22.6 ± 8.3	0.182
Walking speed (m/s)	0.99 ± 0.36	1.07 ± 0.27	0.147

**Abbreviation**: ALM, appendicular lean mass; ALM/BMI, BMI-adjusted ALM; ALM/Wt, weight-adjusted ALM; BDNF, brain-derived neurotrophic factor; BMI, body mass index; ECW, extracellular water; eGFR, estimated glomerular filtration rate; FFM, fat-free mass; hs-CRP, high-sensitivity C-reactive protein; ICW, intracellular water; TBW, total body water.

**Table 3:** Multiple linear regression analyses of the effects of variables on BDNF level.

	Univariable Analysis		Multivariable Analysis	
	β (95% CI)	*p* Value	β (95% CI)	*p* Value
Albumin (mg/dL)	−0.025 (−1.83 × 10^2^, 1.35 × 10^2^)	0.766	−0.046 (−2.05 × 10^2^, 1.18 × 10^2^)	0.596
Creatinine (mg/dL)	−0.134 (−5.49 × 10^2^, 0.48 × 10^2^)	0.100	−0.106 (−5.19 × 10^2^, 1.27 × 10^2^)	0.231
BMI (kg/m^2^)	−0.040 (−0.16 × 10^2^, 0.09 × 10^2^)	0.624	−0.086 (−0.23 × 10^2^, 9.505)	0.410
ALM/BMI (m^2^)	0.052 (−1.34 × 10^3^, 2.62 × 10^3^)	0.526	−0.059 (−3.83 × 10^3^, 2.37 × 10^3^)	0.643
Hand grip (kg)	0.104 (−0.20 × 10^2^, 0.93 × 10^2^)	0.207	0.032 (−0.72 × 10^2^, 0.95 × 10^2^)	0.791
Walking speed (m/s)	0.061 (−9.96 × 10^2^, 2.19 × 10^3^)	0.460	−0.028 (−2.02 × 10^3^, 1.47 × 10^3^)	0.757
ECW/TBW	−0.238 (−9.93 × 10^4^, −2.04 × 10^4^)	0.003	−0.222 (−1.07 × 10^5^, −8.61 × 10^3^)	0.022

**Abbreviation**: CI, confidence interval; BMI, body mass index; ALM, appendicular lean mass; ALM/BMI, BMI-adjusted ALM; ECW, extracellular water; TBW, total body water, BDNF, brain-derived neurotrophic factor.

## Data Availability

The data presented in this study are available on request from the corresponding author.
